# Epidural Brain Metastases in a Patient with Early Onset Pancreatic Cancer: A Case Report and Literature Review

**DOI:** 10.1155/2012/962305

**Published:** 2012-10-17

**Authors:** Aibek E. Mirrakhimov, Farah N. Khan

**Affiliations:** Department of Internal Medicine, Saint Joseph Hospital, 2900 N. Lake Shore, Chicago, IL 60657, USA

## Abstract

We present a case of early onset pancreatic cancer related extra-axial brain metastases. A 46-year-old Caucasian non-Jewish nonobese male with a history of PC diagnosed 3 months ago with metastases to the liver, omentum, malignant ascites, and a history of a pulmonary embolism was admitted to the hospital because of a new onset headache, nausea, and vomiting which started 2 days prior to the encounter. Brain MRI was ordered, which showed acute bihemispheric subdural hematomas and left hemispheric extra-axial heterogeneously enhancing lesions consisting with metastatic disease. The patient was started on ondansentron, metoclopramide, and dexamethasone. The cranial irradiation was started, and the patient's headache and nausea significantly improved. There are only 9 published reports of extra-axial brain metastases related to the pancreatic cancer, whereas our paper is the first such case reported on a patient with epidural metastases and early onset pancreatic cancer.

## 1. Introduction 

Pancreatic cancer (PC) is a common malignant disorder with 43.920 estimated new cases and 37.390 estimated deaths in USA at 2012 [1]. Moreover, it is predicted that PC will be the leading cause of cancer-related mortality in USA by the year 2050. Unfortunately, most cases of PC are recognized too late, when the disease is spread and not amenable to surgical intervention, leading to dismal survival rates [2]. This can be explained by the fact that early PC is minimally symptomatic and lacks specific clinical features. 

Most of the PC cases are diagnosed in patients of age 50 and older [3]. For simplification, the risk factors for PC are divided into environmental and genetic, with smoking being the most studied risk factor. There is an ongoing controversy on whether to include excessive alcohol use as an independent risk factor for PC [4]. Other potential risk factors are chronic pancreatitis [5], obesity [6], and diabetes mellitus (DM), with the latter, in fact, may be a marker and consequence of PC in an elderly patient with a new onset DM [7, 8]. Such risk factors as Helicobacter Pylori infection, hepatitis B virus infection, stomach, and gallbladder surgery are of minor importance and can be found elsewhere. Common gene abnormalities implicated to the occurrence of both sporadic and familial PC are present in Table 1 [9, 10].

As an aggressive tumor, PC metastasizes to the liver and other adjacent organs as well as to the lungs and bones [11], however, only about 0.33% of PCs metastasize to the brain [12]. 

We report a case of an early onset pancreatic adenocarcinoma and epidural brain metastases. The relevant literature will be briefly reviewed and summarized.

## 2. Case Presentation 

A 46-year-old Caucasian non-Jewish nonobese male was admitted to the hospital, because of a new onset headache, nausea, and vomiting. His past medical history is significant for the PC diagnosed 3 months ago after work up of vague abdominal discomfort. The upper gastrointestinal endoscopy was performed at that time and showed few small polyps (nonneoplastic polyps) and mild gastritis. Abdominal CT and ultrasound were done, which showed PC with metastases to the liver and omentum. A biopsy was done which showed pancreatic ductal adenocarcinoma. 

The patient's prior hospital stay was complicated by the development of a pulmonary embolism and small bowel obstruction. It is relevant to note, that the patient never had any mucocutaneous hyperpigmentation, which could be suggestive of Peutz-Jeghers syndrome. The patient received treatment with oxaplatin, 5-fluorouracil, irinotecan, and leucovorin as a chemotherapy for his PC.

During this encounter, the patient described his headache as dull, intractable, and nonradiating. The patient denied fever, skin rash, any history of sick contacts, recent falls, but admitted that he hit his head in the bathroom 1 week prior. The patient denied any history of smoking, alcohol abuse, or use of recreational drugs. It is necessary to note that the patient was discharged from the hospital 8 days prior this admission. 

Family history is significant for colon cancer in his father who was diagnosed at age of 80 and as well as other malignancies on his paternal side (the patient does not remember exactly the relatives affected or the types of cancer). The patient underwent genetic screening for the mutations associated with Lynch syndrome, which were negative (PMS2 sequencing rearrangement, EPCAM rearrangement, MLH1 rearrangement, MSH2 rearrangement, and MSH6 rearrangement analyses). 

On a physical exam, the patient was in moderate to severe distress due to pain and malnourished. His eyes were icteric, equal, and reactive to light and accommodation, with intact extraocular muscles. Fundoscopic exam did not find any retinal abnormality or optic disc swelling. Neck was supple, without palpable thyromegaly and lymphadenopathy. Pulmonary exam was remarkable with decreased air entry at bases bilaterally and respiratory rate of 17 in a minute. Cardiovascular exam showed tachycardic rate with regular rhythm, normal S1/S2, and blood pressure of 116/76. Cranial nerves were grossly normal; muscle strength was decreased diffusely with intact sensation. 

CBC, CMP, urinalysis, and head CT were ordered. His CBC and CMP were unremarkable from the last hospital stay (anemia of chronic disease, mild hyponatremia, and mild increase in alkaline phosphatase). 

**Figure 1:**
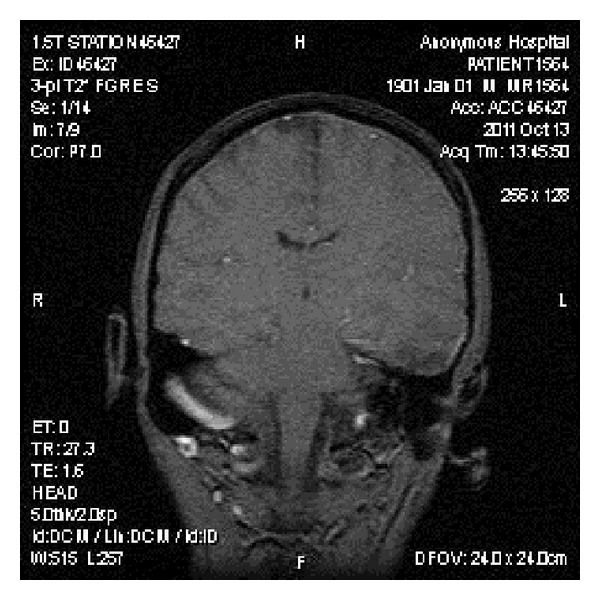
Brain MRI with and without contrast showing bihemispheric subdural hematomas.

**Figure 2:**
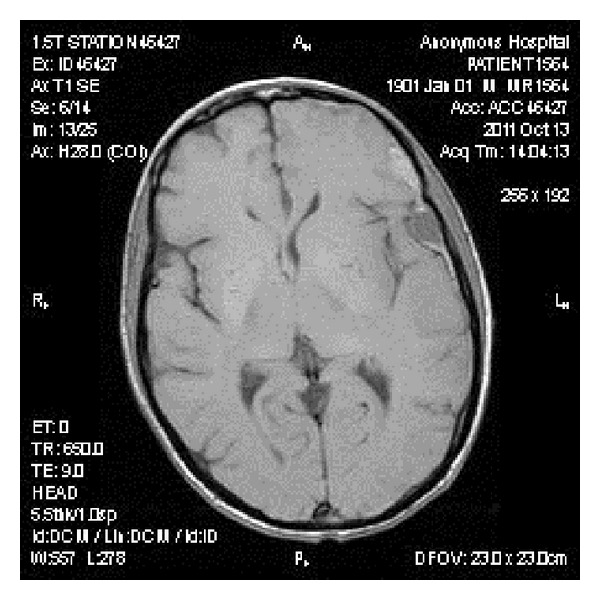
Brain MRI with and without contrast showing a nodular mass reflecting metastatic disease displacing the left inferior frontal gyrus.

CT scan of the head was done which showed acute bihemispheric subdural hematomas with the maximum dimension of 2 centimeters (Figure 1) and neurosurgical consult was obtained, with no surgical intervention being recommended. The patient was started on ondansentron, metoclopramide, and dexamethasone. On the second hospital day, the patient developed syncope, with a generalized tonic-clonic seizure. A repeat CT scan of the head was done, which did not show any new or worsening hematoma. Brain MRI with and without contrast was done, which showed left hemispheric extra-axial heterogeneously enhancing lesions consisting with metastatic disease with the greatest lesion was 4 centimeters in dimension (Figure 2). The patient was started on levetiracetam. The cranial irradiation was started on the third hospital day for 18 sessions with right and lateral fields were exposed to a dose of 37.5 gray in 15 fractions of 250 eGy utilizing 6 MV photons. The patient's headache and nausea significantly improved, and he did not experience any new seizures since then. 

Important to note that patient tolerated radiotherapy well and did not require a treatment break.

The patient died 2.5 months later at home. No autopsy was performed as per family request.

## 3. Discussion

To our knowledge, there are 24 published case reports of PC metastases to CNS with 43 patients studied [13–15]. 7 case reports with 9 patients presented were consistent with extra-axial brain metastases [13]. It is essential to note that most patients will die from their PC prior to development of neurologic symptoms due to CNS metastases [16]. Thus, many patients will be found to have CNS metastases on autopsy or by accidental imaging findings. However, on the other hand, it was speculated that a slightly improved survival rates in patients with PC may increase the number of patients diagnosed with CNS metastases, due to longer survival and a greater time for such metastases to develop [17].

Jacobs and Richland in 1951 [18] and Little et al. in 1974 [19] reported several cases of deceased patients with pancreatic cancer and meningeal carcinomatosis confirmed by autopsy.However, it is important to emphasize that the original reports were done by Scheinker in 1935 and Uspensky in 1943, respectively. Unfortunately, it was impossible for us to find the original manuscripts of these researchers; therefore, we included the aforementioned reports done by Jacobs and Richland [18] and Little et al. [19], which quoted their works.

A case report published in 1972 at New England Journal of Medicine [20] and a case report by Olson et al. [21] reported bilateral subdural hematomas in patients with a primary PC. It is necessary to emphasize that our patient lacks the history of a significant head trauma, and the PC brain metastases might contribute to the development of subdural hematomas in our patient as well.

Leech et al. [22], Galatioto and Savettieri [23], and Ferreira Filho et al. [24] reported cases of meningeal metastases in patients with a primary PC. Thus, our case is the first report on epidural metastases in patient with PC.

Another notable finding was an early onset of PC in our patient and a family history of cancers with colonic adenocarcinoma in his father. 

As outlined above, our patient was tested for the presence of Lynch syndrome, which was negative. Peutz-Jeghers syndrome was not considered due to the absence of classic hyperpigmentation and nonhyperplastic histology of gastric polyps.

Recent data points toward an increased incidence of PC in patients with familial adenomatosis polyposis coli syndrome [25]. However, the most striking pathology of this syndrome is colon cancer, which is uniformly present in affected subjects by the age of 40, which make it unlikely, and this patient has never had any chronic colonic disease. 

As present in Table 1, K-Ras oncogene is implicated in the pathogenesis of the vast majority of pancreatic exocrine carcinoma cases. When, overactive, it provides a potent and continuous stimulus for cell growth and division [8]. It is necessary to emphasize that K-Ras mutations are found in up to half of patients with colon cancer [26]. From a theoretical point of view, it is possible that this abnormality could be present as a germline mutation in our patient; however, medical literature lacks such reports. Hereditary pancreatitis was not considered as well, because it is clinically apparent since childhood [27], and no supporting information was present in this patient.

In addition to the genetic conditions listed, the patient was not tested for other relevant mutations such as TP53, BRCA spectrum, ATM, and CDKN2A. However, based on the clinical presentation, it was unlikely that the patient have had any of them (please see Table 1). However, we did not screen for SMAD4 mutation, which is associated with a greater burden of PC-related metastases (please see Table 1). Theoretically, this abnormality could be present in our patient, since the patient had a metastatically aggressive disease.

Overall, this case report highlights the possibility of epidural CNS metastases related to the PC and the need for further research on the PC genetics to have a better knowledge on the disease pathogenesis and management.

## Figures and Tables

**Table 1:** Common mutated genes implicated in the pathogenesis and occurrence of pancreatic cancer (adapted from [9]).

Name	Chromosome	Normal function	Comment
K-ras	12p	Cell growth and division.	Gain of function mutation. The most common mutation found in PC. Up to 50% of colon cancers harbor the K-ras mutation as well.
TP53	17p	Cell cycle control and regulation of cellular apoptosis.	Loss of function mutation. Implicated in the vast majority of cancers. Abnormal function in Li-Fraumeni syndrome.
CDKN2A	9p	Cell cycle control.	Loss of function mutation. Abnormal function in familial atypical multiple mole melanoma.
SMAD4	18q	Regulation of cell cycle and cell division.	Loss of function mutation. This mutation is associated with a greater burden of PC-related metastases.
BRCA2 and PALB2	13q and 16p, respectively	Regulation of cell cycle.	Loss of function mutations. Abnormal in breast and ovarian cancer syndromes.
STK11/LKB1	19p	Tumor suppressor gene (?)	Loss of function mutation. Abnormal in Peutz-Jeghers syndrome.
MLH1 and MSH2	3p and 2p, respectively	DNA repair and regulation of cell cycle.	Loss of function mutation. Abnormal in Lynch syndrome.
ATM	11q	Regulation of the cell cycle and DNA repair.	Loss of function mutation. Abnormal in Ataxia telangiectasia.
PRSS1 and SPINK1	7q and 5q, respectively.	PRSS1 encodes a trypsinogen and SPINK1 encodes a trypsin inhibitor.	Loss of function mutation for SPINK1 and gain in function mutation for PRSS1. Abnormal in hereditary pancreatitis.
